# Supplementary material for: Content-rich biological network constructed by mining PubMed abstracts
Source: BMC Bioinformatics. 2004 Oct 8;5:147. doi: 10.1186/1471-2105-5-147 (PMC528731; doi:10.1186/1471-2105-5-147)
Supplement: Additional File 2 — The original results of the above study (non-essential files are deleted to keep the file size under the limit set by BMC bioinformatics). [file 1471-2105-5-147-S2.bz2 › chilibotAdditionalFile2/dip05/563ID9374547E10924/html/MST1_MST1R.html]

 


 **MST1** and **MST1R** 
  
Found 69 abstracts in PubMed, retrieved 05.  
 

 What does Google say? 
 PDF only 
| .edu only 

---

**Interactive relationship** (e.g. stimulation, inhibition, etc)

**Stimulatory relationship**- The cytokine Macrophage Stimulating 1  [ **MST1** ]   **MST1**  MSP  [ **MST1** ]  Hepatocyte Growth Factor Like  [ **MST1** ]  is a ligand of the Met related  **MST1**  Receptor  **MST1R**  RON  [ **MST1R** ] .  Ref: 12764615 Dev Genes Evol< MedlineTA>Dev Genes Evol,
- The ligand of RON  [ **MST1R** ]  was identified as macrophage stimulating protein  [ **MST1** ]  MSP  [ **MST1** ] , a member of the plasminogen related growth factor family.  Ref: 12807733 Carcinogenesis< MedlineTA>Carcinogenesis,
**Neutral relationship**- Activation of CR3 mediated phagocytosis by MSP  [ **MST1** ]  requires the RON  [ **MST1R** ]  receptor, tyrosine kinase activity, phosphatidylinositol 3 kinase, and protein kinase C zeta.  Ref: 12773513 J Leukoc Biol, 2003
- We show that MSP  [ **MST1** ]  induced complement mediated phagocytosis requires the RON  [ **MST1R** ]  receptor tyrosine kinase and the alphaMbeta2 integrin, as evidenced by the inability of RON  [ **MST1R** ]  and alphaM peritoneal macrophages to augment phagocytosis of complement coated sheep erythrocytes in response to MSP  [ **MST1** ] .  Ref: 12773513 J Leukoc Biol, 2003

**Non-interactive relationship** (e.g. studied together, co-existance, homology, etc.)

- Oncogenic and invasive potentials of human macrophage stimulating protein  [ **MST1** ]  receptor, the RON  [ **MST1R** ]  receptor tyrosine kinase.  Ref: 12807733 Carcinogenesis< MedlineTA>Carcinogenesis, 2003
- The proto oncogene product Ron  [ **MST1R** ]  is the receptor for macrophage stimulating protein  [ **MST1** ] , a scatter factor that stimulates cell proliferation, prevents apoptosis, and induces an invasive cell phenotype.  Ref: 12766581 Am J Surg Pathol, 2003
- Although  **MST1**  deficient mice are viable,  **MST1R**  is essential in mice before gastrulation for implantation, and is a known oncogene in man.  Ref: 12764615 Dev Genes Evol< MedlineTA>Dev Genes Evol, 2003
- The overexpression of bcl 2, but not p53 in oncocytoma, suggests that the MSP  [ **MST1** ]  Ron  [ **MST1R** ]  system sustains the growth of oncocytoma by opposing apoptosis.  Ref: 12766581 Am J Surg Pathol, 2003
- Ron  [ **MST1R** ] , the receptor tyrosine kinase RTK for the macrophage stimulating protein  [ **MST1** ]  MSP  [ **MST1** ] , activates multiple signaling pathways by recruiting several positive regulators to a multifunctional docking site.  Ref: 12802274 Oncogene, 2003
